# Dysferlin-Peptides Reallocate Mutated Dysferlin Thereby Restoring Function

**DOI:** 10.1371/journal.pone.0049603

**Published:** 2012-11-20

**Authors:** Verena Schoewel, Andreas Marg, Severine Kunz, Tim Overkamp, Romy Siegert Carrazedo, Ute Zacharias, Peter T. Daniel, Simone Spuler

**Affiliations:** 1 Muscle Research Unit, Experimental and Clinical Research Center, a joint cooperation between the Charité Medical Faculty and Max Delbrück Center for Molecular Medicine, Berlin, Germany; 2 Clinical and Molecular Oncology, University Medical Center Charité, Campus Berlin-Buch, Berlin, Germany; University of Edinburgh, United Kingdom

## Abstract

Mutations in the dysferlin gene cause the most frequent adult-onset limb girdle muscular dystrophy, LGMD2B. There is no therapy. Dysferlin is a membrane protein comprised of seven, beta-sheet enriched, C2 domains and is involved in Ca^2+^dependent sarcolemmal repair after minute wounding. On the protein level, point mutations in *DYSF* lead to misfolding, aggregation within the endoplasmic reticulum, and amyloidogenesis. We aimed to restore functionality by relocating mutant dysferlin. Therefore, we designed short peptides derived from dysferlin itself and labeled them to the cell penetrating peptide TAT. By tracking fluorescently labeled short peptides we show that these dysferlin-peptides localize in the endoplasmic reticulum. There, they are capable of reducing unfolded protein response stress. We demonstrate that the mutant dysferlin regains function in membrane repair in primary human myotubes derived from patients’ myoblasts by the laser wounding assay and a novel technique to investigate membrane repair: the interventional atomic force microscopy. Mutant dysferlin abuts to the sarcolemma after peptide treatment. The peptide-mediated approach has not been taken before in the field of muscular dystrophies. Our results could redirect treatment efforts for this condition.

## Introduction

Dysferlin is a type II membrane protein in the ferlin family with a short extracellular 12 amino-acid domain. Dysferlin contains seven C2 domains and is involved in Ca^2+^dependent membrane repair after sarcolemmal microinjuries [1], [2]. Limb-girdle muscular dystrophy 2B (LGMD2B) is the most prominent dysferlinopathy caused by mutations in the dysferlin (*DYSF*) gene [3], [4]. Young adults are affected and become wheel-chair bound within 15 years. Other dysferlinopathies are Miyoshi myopathy, anterior-tibial myopathy, idiopathic hyperCKemia, and rare congenital forms. There is no treatment. *DYSF* is a large gene with 55 exons encoding a 2080 amino-acid protein. More than 1000 *DYSF* mutations have been described [5], [6]. They are distributed evenly over the entire length of the gene without mutational hotspots.

At least a third of the *DYSF* mutations are missense mutations. We and others have demonstrated that *DYSF* missense mutations cause dysferlin aggregation and misfolding, amyloid formation, and subsequent degradation by the endoplasmic reticulum-associated protein degradation machinery (ERAD) [7], [8], [9]. Protein misfolding is the cause of numerous progressive hereditary and acquired diseases including Alzheimer's dementia, Parkinson's disease, and cystic fibrosis. Research directed towards protein refolding and prevention of premature degradation in the endoplasmic reticulum (ER) is intensively studied [10], [11]. Dysferlinopathy is the first muscular dystrophy where faulty protein aggregation was established. We asked whether mutant dysferlin trapped within the ER could be redirected to the sarcolemma and whether mutant dysferlin would be functional at this site. We selected specifically designed dysferlin-peptides coupled to the cell-penetrating peptide TAT (trans-activator of transcription from human immunodeficiency virus type 1) and demonstrate that these peptides support mutant dysferlin to relocate and to regain proper function.

## Results

### Dysferlin-peptides Cause *DYSF* Missense Mutants to Relocate to the Sarcolemma in C2C12 Cells

**Figure 1:**
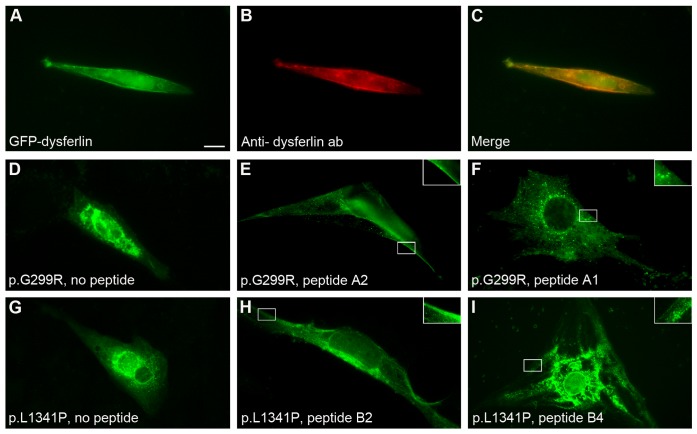
Dysferlin-peptides redirect mutant dysferlin in transfected C2C12 cells. C2C12 cells were transfected with either GFP-tagged wildtype human dysferlin cDNA or missense-mutated dysferlin cDNA *DYSF* p.G299R or p.L1341P. Transfected cells were treated with TAT-labeled dysferlin-peptides corresponding to the mutation. (**A–C**) Wildtype human dysferlin-GFP tagged. (**A**) Dysferlin-GFP. (**B**) Localization of dysferlin is confirmed by immunostaining using an anti-dysferlin ab. (**C**) merge. (**D–F**) *DYSF* p.G299R-GFP tagged. Experiment performed 14×. (**D**) No peptides added. GFP-dysferlin is not expressed at the plasma membrane. (**E**) Peptide A2 (10-mer) added corresponding to *DYSF* p.G299R. GFP-dysferlin relocalizes to sarcolemmal sites. (**F**) Peptide A1 corresponding to WT dysferlin added. Distribution of dysferlin is granular but not expressed at the sarcolemma. (**G–I**) *DYSF* p.L1341P-GFP tagged. Experiment performed 12×. (**G**) No peptides added. (**H**) Peptide B2 (10-mer) corresponding to *DYSF* p.L1341P supports reallocation of dysferlin to the sarcolemma. (**I**) Peptide B4 (15-mer) corresponding to *DYSF* p.L1341P. 10mer peptides carrying the corresponding mutation were most effective. Bar: 10 µm. Inserts represent enlarged boxed areas.

We selected two defined *DYSF* missense mutations for our experimental design: *DYSF* p.G299R and *DYSF* p.L1341P. The consequences of these mutations at the protein level have been shown previously [6]. Both mutations lead to the absence of dysferlin at the sarcolemma and to protein aggregation within the ER. These variants were cloned into the pcDNA4/TO vector containing the full-length human *DYSF* cDNA-GFP-tagged. C2C12 murine muscle cells were transfected with wildtype (WT) or mutant dysferlin. Transfection with WT dysferlin resulted in membrane staining (Fig. 1A–C); however, the mutants as expected, aggregated within the cells (Fig. 1D, G). We next explored the effect of peptides specifically designed to resemble the respective *DYSF* mutations. We coupled 10mer and 15mer dysferlin-peptides to the human immunodeficiency virus transactivator protein at their N-terminal ends (Table 1 and Fig. S1). Eleven hours after last addition of TAT-labeled dysferlin-peptides to C2C12 cells, we detected dysferlin at sarcolemmal sites. This effect was most efficient when the shortest peptides were used that contained the mutated amino acid (Fig. 1E, H). Longer peptides (15mer) caused a weaker membranous redistribution (Fig. 1I and Fig. S2B); peptides reflecting the wildtype sequence led to a dispersion of otherwise accumulated dysferlin but we were not able to detect dysferlin at the muscle membrane (Fig. 1F and Fig. S2D). All experiments were also performed in C2C12 cells harboring a permanent knockdown of the dysferlin gene (not shown).

**Table 1:** Dysferlin-derived peptides.

Nomenclature	Description	Sequence of TAT-labeled dysferlin-peptides
***DYSF*** ** p.293–305: LRTDALLGEFRMDVG**		
**A1**	10mer, analogous to wildtype	YGRKKRRQRRR- DALLGEFRMD- amid
**A2**	10mer, analogous to mutant	YGRKKRRQRRR- DALLREFRMD- amid
**A3**	15mer, analogous to wildtype	YGRKKRRQRRR- LRTDALLGEFRMDVG- amid
**A4**	15mer, analogous to mutant	YGRKKRRQRRR- LRTDALLREFRMDVG- amid
***DYSF*** ** p.1434–1448: IEILAWGLRNMKSYQ**		
**B1**	10mer, analogous to wildtype	YGRKKRRQRRR- LAWGLRNMKS- amid
**B2**	10mer, analogous to mutant	YGRKKRRQRRR- LAWGPRNMKS- amid
**B3**	15mer, analogous to wildtype	YGRKKRRQRRR- IEILAWGLRNMKSYQ- amid
**B4**	15mer, analogous to mutant	YGRKKRRQRRR- IEILAWGPRNMKSYQ- amid
**Fluorescent B2 peptide**	10mer, analogous to mutant	YGRKKRRQRRR- C(ATTO-495-ME)-LAWGPRNMKS- amid
**Control**		
**Nonsense peptide**	No analogy to *DYSF* sequence	YGRKKRRQRRR- SMLAPWRGNK- amid

### Dysferlin-peptides Cause Reallocation of *DYSF* Missense Mutants in Primary Human myotubes

**Figure 2:**
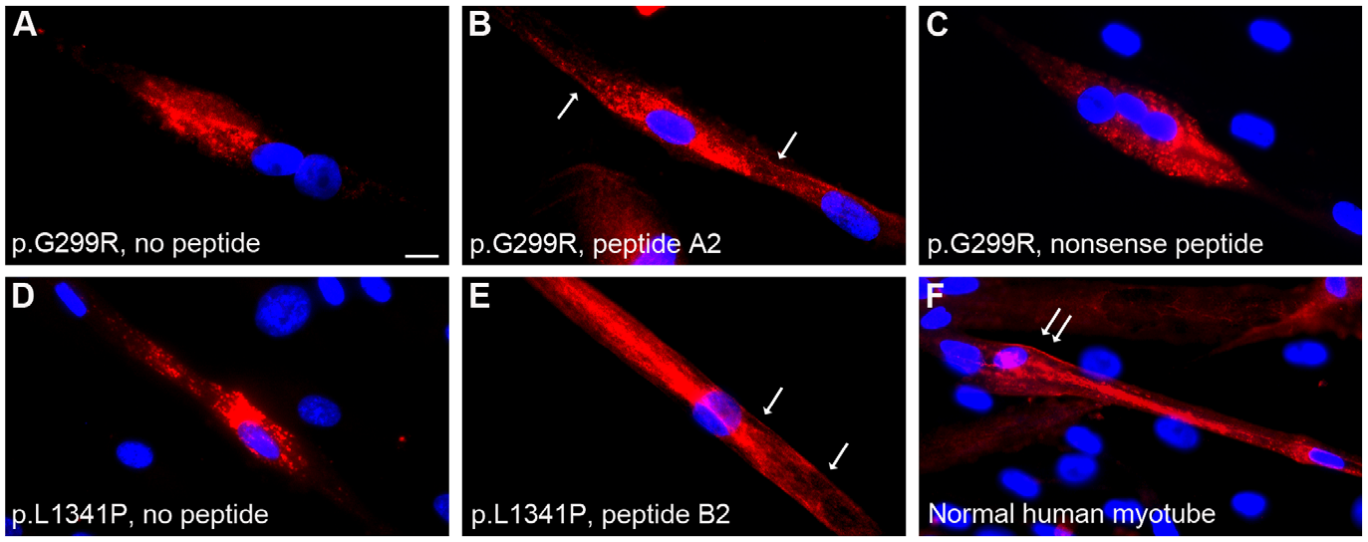
Dysferlin-peptides redirect mutant dysferlin to the sarcolemma in primary human myotubes. Primary human myotubes carrying dysferlin missense mutations were treated with the TAT-labeled dysferlin-peptides. Dysferlin was detected by anti-dysferlin ab. Nuclei are stained with Hoechst. Missense mutated dysferlin aggregates within the myotubes (**A, D**). After treating human myotubes with 10mer peptides (**B, E**) harboring the missense mutation mutant dysferlin can be localized at sarcolemmal sites whereas nonsense peptides (**C**) do not elicit this effect. (**A–C**) Primary human myotubes expressing *DYSF* p.G299R. Experiment performed 9x. (**A**) No peptides added. (**B**) Peptide A2 (10-mer) added corresponding to *DYSF* p.G299R. (**C**) Nonsense peptide added (control). (**D–E**) Primary human myotubes harboring the dysferlin mutation p.L1341P. Experiment performed 7x. (**D**) No peptides added. (**E**) Peptide B2 (10-mer) added corresponding to *DYSF* p.L1341P. (**F**) Sarcolemmal dysferlin localization in a normal human myotube. Bar: 10 µm. Arrows indicate reallocated dysferlin to sarcolemmal sites.

We next tested whether or not TAT-labeled dysferlin-peptides would elicit an effect on primary human myotubes obtained from patients with *DYSF* p.G299R and *DYSF* p.L1341P. Missense mutated dysferlin accumulates within the myotubes (Fig. 2A and D, Fig. S3) and does not localize to the sarcolemma as it can be seen in multinucleated human muscle cells from normal controls (Fig. 2F). Similar to the results seen in C2C12 cells, addition of TAT-labeled specific peptides to dysferlin-deficient human myotubes resulted in translocation of mutant dysferlin to the sarcolemma (Fig. 2B and E, Fig. S3). Again, 10-mer peptides containing the corresponding mutation were most effective. Peptides corresponding to the wildtype sequence or nonsense peptides (Fig. 2C, Fig. S3) did not show these effects on dysferlin localization. Thus, the short peptides derived from mutant dysferlin and coupled to TAT are effective in retargeting dysferlin to the sarcolemma.

### Missense Mutant dysferlin is Functional after Reallocation

**Figure 3:**
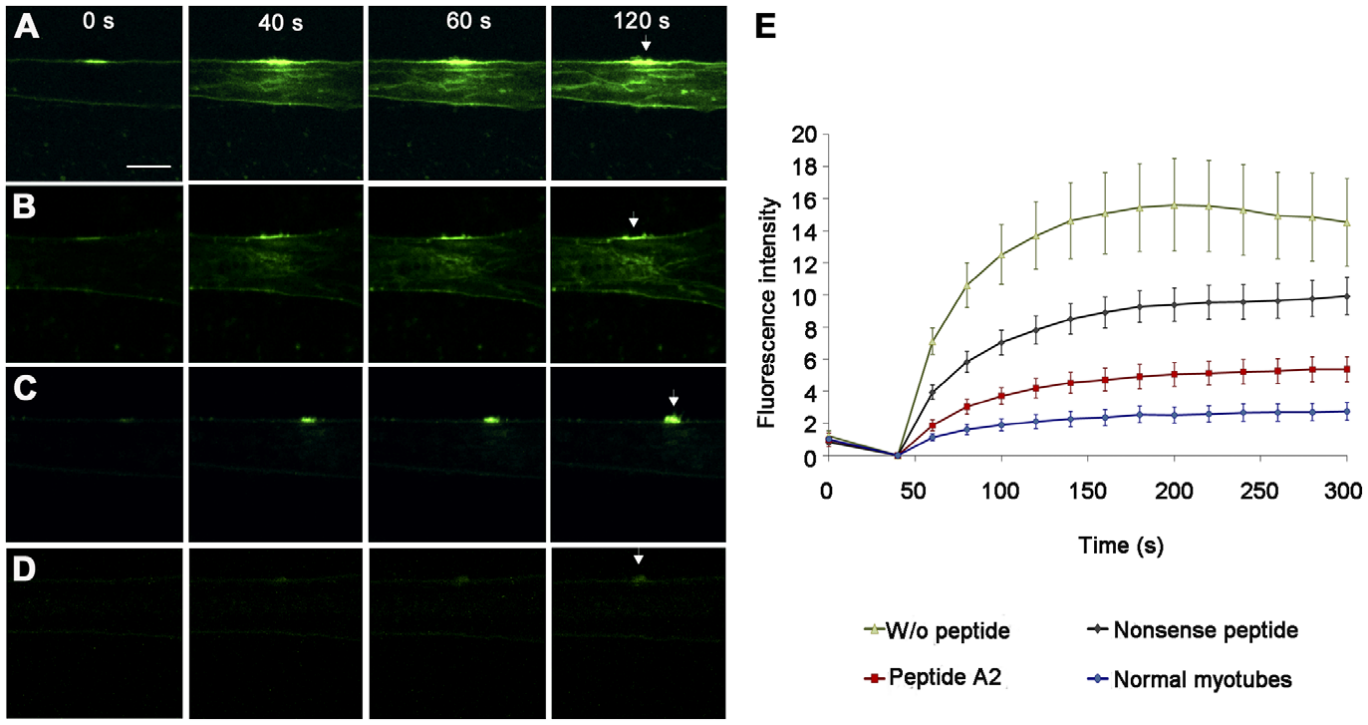
Impaired membrane repair in dysferlin deficient myotubes was rescued by specific peptides: Laser wounding. Fluorescent dye influx (FM1-43) in human myotubes harboring *DYSF* p.G299R or in normal human myotubes is shown at the time of laser wounding (0 seconds) and 40, 60 and 120 seconds thereafter. Myotubes were wounded by irradiating a 2.5×2.5 µm boundary area of the plasma membrane. (**A**) *DYSF* p.G299R, no peptide. (**B**) *DYSF* p.G299R, nonsense peptide added. (**C**) *DYSF* p.G299R, corresponding mutant peptide A2 added. (**D**) Normal human myotubes, no peptides. Without peptides or after incubation with nonsense peptide FM1-43 dye rapidly spreads within the *DYSF* p.G299R human myotubes. After treatment with specific peptide A2 distribution of FM1-43 is similar to normal myotubes. Arrows indicate the wounded area of the plasma membrane. Scale bar: 10 µm. (**E**) Quantification of fluorescence intensity (FM1-43) before (0 seconds) and after laser wounding. Data represent mean ± SEM, n = 6/group. See also video S1, S2, S3, S4.

We next tested the function of mutant dysferlin reallocated with TAT-labeled dysferlin-peptides by two independent methods. First, we performed laser wounding of the sarcolemma in primary human myotubes. Due to the impaired membrane repair ability dysferlin-deficient myofibers show influx of fluorescent dye into the myotube [2]. We adapted the method to primary human myotubes. Because of the smaller size of the myotubes as compared to murine intact muscle fibers the wounding area was only 2.5×2.5 µm. Compared to normal human myotubes (Fig. 3D and E, video S4) dye influx was significantly increased in dysferlin-mutants (Fig. 3A and E, video S1). TAT-labeled dysferlin-peptides harboring the mutation significantly improve the ability of the sarcolemma to reseal (Fig. 3C and E; video S3). Non-specific peptides also elicited some effect on membrane resealing; however, this effect did not reach statistical significance (Fig. 3B and E, video S2).

**Figure 4:**
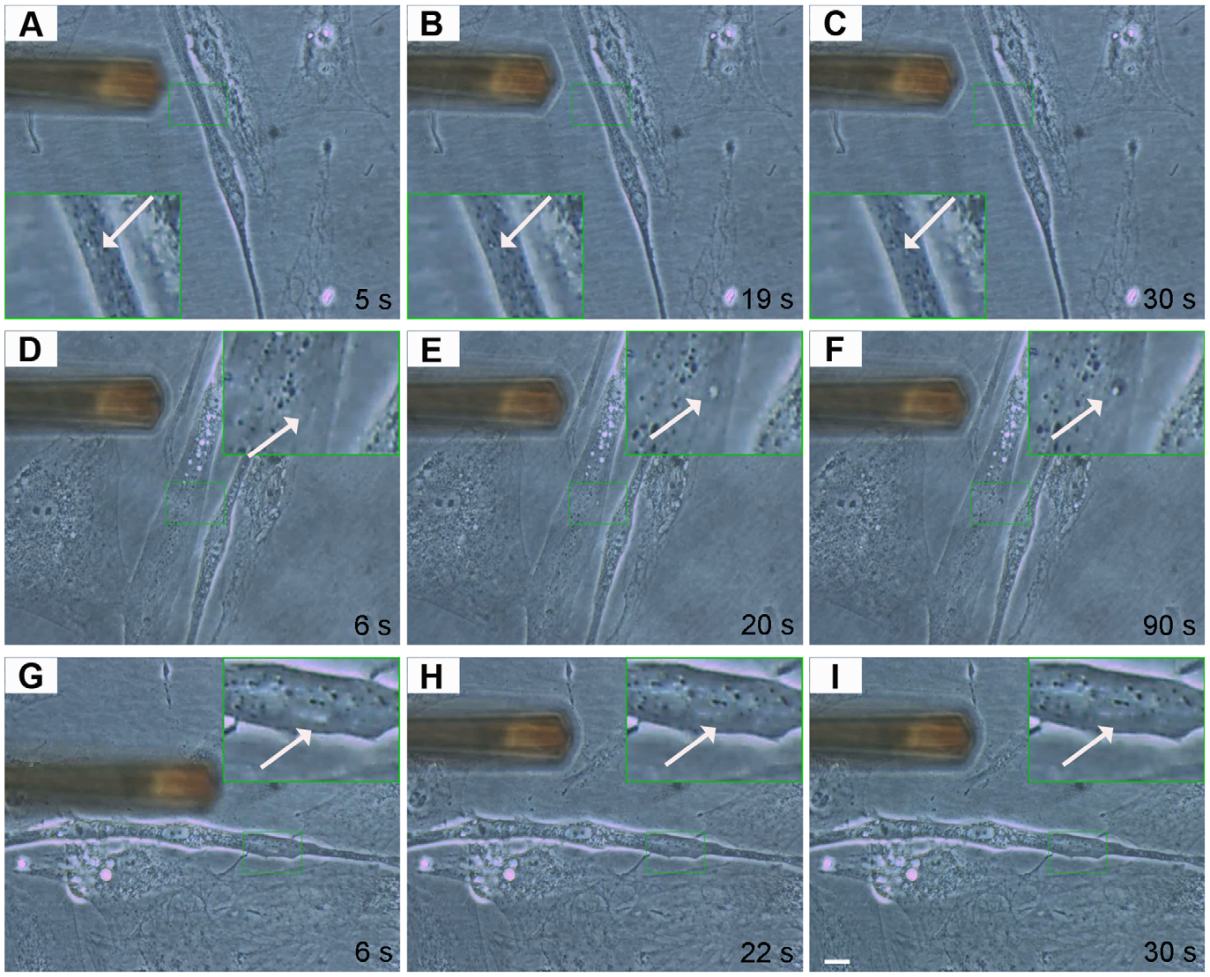
Specific peptides cause functional recovery in dysferlin-deficient myotubes: Interventional atomic force microscopy. Mechanical sarcolemmal wounding was induced by atomic force microscopy. In all experiments 2 µm longitudinal lesions were set. Arrows indicate the lesion site. The instrument shown is the cantilever used for membrane wounding. (**A–C**) Normal human myotube 5, 19 and 30 seconds after wounding. The lesion is hardly detectable and closes rapidly. (**D–F**) *DYSF* p.L1341P primary human myotube 6, 20 and 90 sec after wounding. The lesion continuously increases in size. (**G–I**) *DYSF* p.L1341P primary human myotube treated with corresponding B2 peptide 6, 22 and 30 seconds after wounding. The lesion disappears rapidly. Scale bar: 10 µm. See also video S5, S6, S7.

We applied a second method to wound myotubes at the single-cell level. We used atomic force microscopy to induce and visualize membrane wounding and repair in normal and dysferlin-mutant human myotubes. *DYSF* p.L1341P myotubes, untreated and treated with TAT-labeled dysferlin-peptides, were wounded by applying a 2 µm longitudinal cut with a force of 40 nN with a CSC37 cantilever (Fig. 4 and video S5, S6, S7). The size of lesions corresponded to the description of earliest events of membrane disruptions in dysferlinopathies [12]. We detected membrane healing in normal (Fig. 4A–C, video S5) and also in dysferlin-peptide treated LGMD2B (Fig. 4G–I, video S7) myotubes. Untreated dysferlin-mutant myotubes did not exhibit repair (Fig. 4D–F, video S6).

### TAT-labeled Mutant dysferlin-peptides Localize to the ER

**Figure 5:**
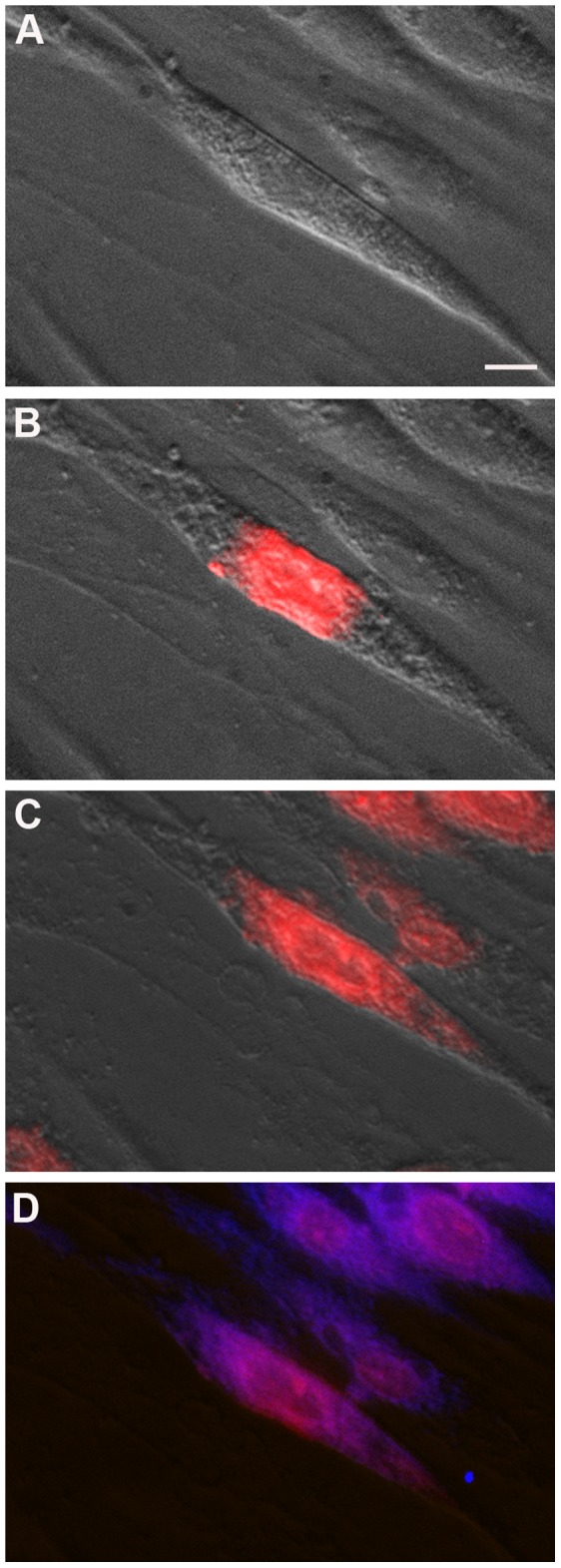
TAT-labeled dysferlin-peptides in primary human myotubes localize to the ER. Peptide B2 corresponding to *DYSF* p.L1341P was labeled with ATTO-495-ME fluorescent dye (red) and added to *DYSF* p.L1341P human myotubes. (**A**) *DYSF* p.L1341P human myotube immediately after addition of ATTO-495-ME-peptide B2 to *DYSF* p.L1341P human myotubes, (**B**) after 8 minutes and (**C**) after 4 hours. (**D**) Immunostain using anti-calnexin ab (blue). Perfect co-localization of ATTO-495-ME-peptide B2 and ER marker calnexin. No dysferlin-peptide detected at the sarcolemma. Bar: 10 µm. See also video S8.

To investigate the mechanism elicited by the dysferlin derived peptides, we monitored their fate after administration to myotubes. Peptide B2, which corresponds to the *DYSF* p.L1341P mutation, was fluorescently labeled with ATTO 495-ME (YGRKKRRQRRR-C(ATTO-495-ME)-LAWGPRNMKS-amid) and continuously tracked within the myotube for 14 hours in a life cell imaging setup. The fluorescent TAT-labeled dysferlin-peptide co-localized with calnexin, a marker of the ER. However, this construct never appeared at the cell membrane (Fig. 5 and video S8). Thus, mutant dysferlin-peptides do not migrate to the sarcolemma, but instead localize to the ER. We explored the possibility that the mutant dysferlin peptide might directly bind dysferlin. We performed protein-binding studies between the recombinant C2E domain of dysferlin that contained the *DYSF* p.L1341P mutation and the corresponding wildtype or mutant dysferlin peptides by surface plasmon resonance analysis. Binding between peptides and recombinant dysferlin was not observed (Fig. S4).

### ER Stress is Reduced by Treating *DYSF* p.G299R Expressing myotubes with TAT-Labeled Mutant dysferlin-peptides

**Figure 6:**
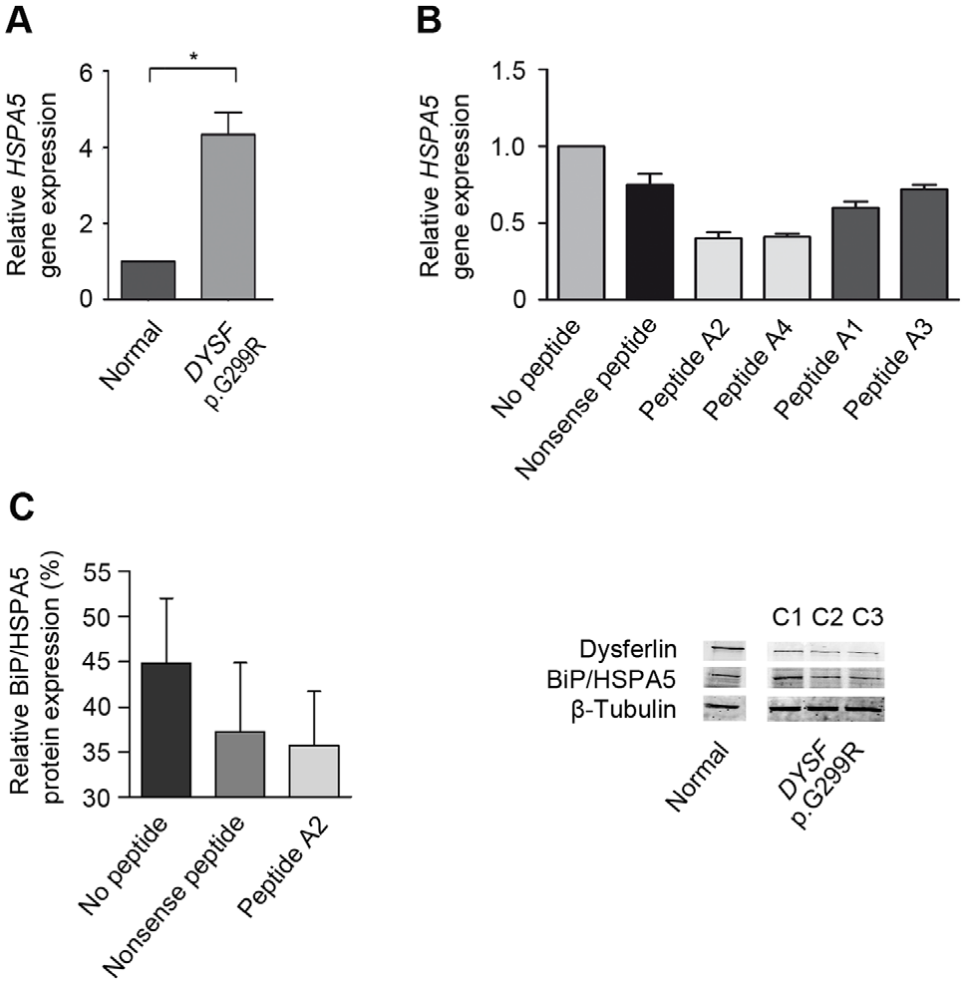
TAT-labeled dysferlin-peptides reduce the expression of the ER stress sensor BiP. (**A**) In *DYSF* p.G299R human myotubes *HSPA5* gene expression is significantly up-regulated. Data represent median + SEM, n = 9/group. (**B and C**) Mutant peptides decrease *HSPA5* gene expression in *DYSF* p.G299R human myotubes most effectively (RT-PCR). Consequently, treatment with specific peptide A2 reduces BiP protein expression. C1: no peptides added; C2: addition of nonsense peptide; C3: treatment with specific peptide A2. The nonsense peptide serves as a control and its amino-acid sequence has no analogy to dysferlin. Data represent median + SEM, n = 3/group. Fig. S5 provides additional information on statistics.

Protein misfolding may lead to the activation of the unfolded protein response (UPR) and ER stress. Indeed, we found a significant up-regulation of *HSP5A* mRNA encoding for the ER stress sensor BiP in myotubes harboring the *DYSF* p.G299R mutation compared to normal controls (Fig. 6A). The immunoglobulin heavy chain binding protein BiP (78 kDa glucose-regulated protein, GRP78, HSPA5) is one of the key representatives of the heat shock protein family hsp 70 and was the first namely identified member of the group of ER chaperones [13]. BiP mainly retains misfolded proteins in the ER [14] and is jointly responsible for transferring misfolded proteins through the ER membranes back to the cytosol. Indeed, a complex of EDEM, ERdj5 and BiP prepares and provides terminally misfolded proteins for proteasomal degradation [15]. In case of accumulation of misfolded proteins in the ER, the *HSPA5* gene transcription coding for the protein BiP is up-regulated [16] assigning BiP the role of an ER stress sensor [17], [18], [19], [20]. After treatment with the dysferlin-derived mutant peptides we could detect a down-regulation of BiP mRNA expression as well as a reduction on protein level (Fig. 6B–C, Fig. S5C). Moreover, the relative gene expression of ATF6 is down-regulated after treatment. AFT6 is a key transcription factor which transmits ER stress signals to the nucleus inducing expression of ER stress-response genes [21], [22]. In addition, we observed a down-regulation of gene expression of the translation attenuator PERK (*EIF2AK3*) [23], [24] as well as CHOP (*DDIT3*), which induces ER stress mediated apoptosis [25], [26], [27] (Fig. S5). Thus, specific peptide treatment decreases ER stress in human myotubes expressing *DYSF* missense mutants.

## Discussion

For the first time, a peptide-mediated therapy approach has been taken in the field of muscular dystrophies redirecting a mutant protein to the plasma membrane thereby restoring function. We and others have demonstrated that missense mutated dysferlin causes toxic “gain-of-function” through development of amyloid fibrils and activation of ERAD, as well as loss of function due to failure of dysferlin to anchor into its regular site of action. We show that by adding dysferlin-peptides to the cell-penetrating TAT peptide, constructs can enter the ER, reduce UPR stress and release missense mutant dysferlin to the sarcolemma. Moreover, this mutant dysferlin is capable of regaining function. Dysferlinopathy is the only muscular dystrophy so far where a contribution of protein misfolding to disease pathology has been described. However, in other muscle disorders like myofibrillar myopathies, inclusion body myopathies, nemalin myopathies, and FHL-1 mutation-associated muscle disorders protein misfolding has been implied. All these diseases could be candidates that might benefit from rectifying faulty protein aggregation [28], [29], [30], [31].

Due to their low membrane permeability, peptides were long considered to be of minimal therapeutic value. However, the discovery of cell-penetrating peptides (CPPs) that readily cross lipid bilayers has changed this state-of-affairs [32]. These peptides are rich in cationic amino acids and are only about 10 to 16 amino acids long. Today, many different CPPs have been described; TAT, an HIV-peptide, has been the most extensively studied. We demonstrate that uptake into mature myotubes is rapid and efficient (Fig. 5). *In vivo*, the use of CPP-coupled peptides is more complicated because CPPs lack cell-specificity. Strategies to overcome this problem have been addressed and are based on the physiological or biological features of the targeted organ. To test possibilities specifically targeting skeletal muscle *in vivo* by CPP-coupled peptides will be both interesting and formidable. The fact that TAT-peptide cargo associates and co-localizes with markers of caveolar uptake, may point towards a way to use muscle-specific caveolae as targets [33], [34].

The laser-wounding assay has been used earlier to document the functional role of dysferlin at the cell membrane [2], [35]. We also developed an independent wounding assay that allowed us to follow the fate of wounds on a single-cell level [36]. We used atomic force microscopy (AFM) to damage the sarcolemma mechanically and to observe repair kinetics. In atomic force microscopy, a cantilever is used to scan living or solid surfaces on nanoscale; however, interventions at the cell surface are also possible as shown here [37], [38]. Both assays demonstrate convincing repair of sarcolemmal wounds after treating human myotubes harboring a dysferlin missense mutation with TAT-labeled dysferlin-peptides. In the future, interventional atomic force microscopy may become a valuable tool to dissect sarcolemmal wounding and repair events on molecular level- possibly identifying new mechanisms or proteins in a growing family of molecules associated with membrane repair.

We are confident that dysferlin relocated to the membrane after treatment with TAT-labeled peptides because of restored function. However, sarcolemmal insertion of endogenous mutant dysferlin would only unambiguously be depicted by detecting the extracellular C-terminus on non-permeabilized myotubes. Because the extracellular portion of dysferlin is only 12 amino acids in length the generation of an antibody against this epitope has not been successful.

Many different strategies are currently being considered to deal with protein misfolding in various disorders. Physiological and pharmacological chaperones and small molecules derived from large library screens could be effective. In this context those compounds become of increasing interest which act with higher specificities and therefore with reduced toxicity [39]. In missense-mutated dysferlin we have applied a membrane repair assay, which is labor and time intensive, thus unsuitable for high throughput screen. However, in searching for strategies to intervene with misfolded dysferlin, we identified similarities to approaches used to unfold mutated cystic fibrosis transmembrane regulators (CFTR), the membrane protein affected in cystic fibrosis. In a mechanism called transcomplementation, fragments of the wild-type CFTR protein can rescue the functionally intact chloride channel at the cell membrane. The restoration process was validated *in vitro* and in cystic fibrosis mice *in vivo* [40], [41]. The mechanism of peptide-mediated relocalization is still unknown.Instead of delivering cDNA vector constructs encoding for truncated proteins to the targeting tissue, here we directly administered the short peptide itself avoiding the need of any viral gene delivery approach. Our results confirm the cellular localization of the peptides in the ER, but in our hands best results were achieved with therapeutic peptides harboring the mutation and not reflecting the wildtype sequence.

Recently, it was shown, that the isolated C2B to C2G domain as well as the transmembrane region of dysferlin are capable of binding to their homologous partners. These interactions form the total dysferlin protein to a homodimer *in vivo* [42]. We investigated two missense mutations located in different C2 domains of the dysferlin protein structure: the C2B and C2E domain (Fig. S1). Here, we show strong evidence, that the specific peptides reflecting the dysferlin sequence itself allow some dysferlin harboring the amino acid exchange to escape protein accumulation and degradation to fulfill their task at the plasma membrane. This might lead to the hypothesis, that the specific peptides may transiently be capable of stabilizing the missense mutated proteins during folding in the ER. Supportively, the primary up-regulation of the ER stress sensor BiP in human myotubes expressing misfolded dysferlin can be reversed by treatment with dysferlin mutant peptides (Fig. 6). This would be consistent with the reduction of the amount of misfolded proteins within the ER by dysferlin-peptide treatment. Consequentially, gene expression of the ER stress mediator proteins ATF6 and PERK were likewise down-regulated after treatment as well as the expression of CHOP, an inducer of ER stress-mediated apoptosis (Fig. S5). The amelioration of ER stress-induced dysfunction may additively contribute to the restoration of the cellular function. Apparently, the amount of dysferlin at the sarcolemma required for normal sarcolemmal wound repair is only around 10% [43], suggesting that a therapeutic approach might even be effective despite an incomplete rescue.

Missense mutated dysferlin has been lately shown to may escape protein degradation by unspecific blocking of the ubiquitin-proteasome system [44]. Bortezomib has been approved for the therapeutically treatment of multiple myeloma and mantle cell lymphoma taking advantage of its primary effect on cells with high proliferation rate and therefore high protein turnover inducing cell cycle arrest and apoptosis [45]. The application regime is limited to the development of adverse events due to the unspecific affection of other organ systems and the overall activation of the unfolded protein response [46], [47], [48], [49], [50]. Taking this into account, the long-term treatment of chronic diseases ideally relies on the specific targeting of the particular misfolded candidate protein and the stabilization of cellular homeostasis by reducing ER-stress related dysfunction. The dysferlin-derived peptide treatment provides a promising approach to fulfill these criteria. Imaging studies after administration of labeled peptides in an animal model would provide further insights regarding the systemic tissue distribution and functional specificity.

We conclude, that the TAT-labeled dysferlin-specific peptides promote mutant dysferlin to reallocate within the cell and to regain function. The peptides function in the ER by interfering with the accumulation of misfolded dysferlin and thereby reducing ER stress in patients’ myotubes.

## Materials and Methods

### Ethics Statement: Patients and Patient Material

Muscle biopsy specimens from patients with LGMD2B (dysferlin mutations) were obtained for diagnostic purposes and from normal controls were obtained during hip surgery (Ethical approval EA1/203/08). All studies were performed according to the declaration of Helsinki and were approved by the local ethical committee (Ethics Committee – Charité, University Medicine Berlin). Written informed consent was obtained from all subjects. The patients with dysferlinopathy were affected by the following dysferlin mutations: homozygous c.4022T>C (patient 1), compound heterozygous c.855+1delG/c.895G>A (patient 2).

### Cells and Cell Culture

C2C12 cells were obtained from DSMZ (Braunschweig, Germany). Dysferlin-deficient C2C12 cells were a gift from Dr. Robert Brown (University of Massachusetts School of Medicine, Worcester, USA) [51]. Dysferlin-deficient C2C12 cell line required puromycin (1.5 µg/ml) in the culture medium. Most experiments on human myotubes were performed with primary human muscle cells isolated from healthy probands and from patients with LGMD2B: *DYSF* c.855+1delG and c.895G>A; p.G299R; homozygosity for *DYSF* c.4022T>C; p.L1341P. Muscle specimens were placed in 30.2 mM HEPES containing 130 mM NaCl, 3 mM KCl, 10 mM D-Glucose and subsequently digested in 254 U/ml Collagenase CLS II (Biochrom AG, Berlin, Germany), 100 U/ml Dispase II (Roche, Grenzach-W´yhlen, Germany) and trypsin/EDTA at 37°C for 45 minutes. Cells were grown in skeletal muscle cell growth medium (Promocell, Heidelberg, Germany) supplemented with 10% fetal bovine serum (Lonza, Cologne, Germany), 2.72 mM glutamine (GlutaMAX™) and gentamicin (400 µg/ml) (Gibco, Paisley, UK). Myoblasts were purified using anti-CD56 ab-coated magnetic beads (Miltenyi Biotech, Bergisch Gladbach, Germany). Differentiation into myotubes was induced in DMEM containing 2% horse serum (fusion media). The experiments shown in Fig. S3B were performed on immortalized human muscle cells endogenously expressing the *DYSF* p.L1341P mutation [52].

### Dysferlin-peptides

Corresponding to wildtype dysferlin and dysferlin mutations *DYSF* p.G299R and *DYSF* p.L1341P 10 and 15mer peptides were synthesized and coupled to TAT (YGRKKRRQRRR) (Biosynthan, Berlin, Germany) (Tab.1 and Fig. S1). Peptide B2 was fluorescently labeled with ATTO 495-ME. A nonsense peptide without analogy to dysferlin was also generated to serve as control.

### Plasmids and Transfection, Peptide Treatment

The pcDNA4/TO vector (Life Technologies GmbH/Invitrogen, Darmstadt, Germany) containing full-length human *DYSF* cDNA-GFP-tagged was a gift from Dr. Steven Laval (Newcastle Upon Tyne, UK). *DYSF* c.895G>A leading to *DYSF* p.G299R and *DYSF* c.4022T>C leading to *DYSF* p.L1431P were cloned into the vector by site-directed mutagenesis. For transfection, C2C12 cells were maintained in fusion media for four days and then transfected with Lipofectamine™ and Plus Reagent™ (Invitrogen, Karlsruhe, Germany). 6 and 23 hours after transfection cells were treated with TAT-labeled dysferlin-derived peptides (10 µM). Cells were fixed 34 hours after transfection.

For the dysferlin-peptide treatment of human primary myotubes cells were maintained in fusion media for seven days. 32, 20 and 8 hours before fixation cells were treated with TAT-labeled specific peptides (10 µM).

### Immunofluorescence and Western Blotting

HAMLET anti-dysferlin monoclonal antibody (ab) was purchased from Novocastra (Newcastle Upon Tyne, UK). Monoclonal mouse anti-calnexin ab was acquired from Acris (Hiddenhausen, Germany). Texas red pre-labeled WGA (EY Laboratories, San Mateo, CA, USA) was used as marker for the basal lamina. Nuclei were stained using Hoechst 33342 (Invitrogen). Monoclonal mouse anti-BiP/GRP78 antibody was obtained from BD Biosciences (Heidelberg, Germany). Rabbit polyclonal antibody against tubulin served as a loading control (Abcam, Cambridge UK). Immunofluorescence and Western blotting were performed according to standard protocols. Protein bands were quantified by Adobe Photoshop CS4. Immunofluorescence images were collected using the Leica DMI6000 (Leica Microsystems, Wetzlar, Germany) or the Zeiss LSM 700 confocal microscope (Carl Zeiss Microscopy GmbH, Germany) both equipped with a 63× glycerin immersion lens. Digital images were processed using the LAS AF software (Leica) or the Zeiss LSM ZEN software 2010 (Carl Zeiss). Images were assembled using Photoshop CS4 (64 bit).

### Life Cell Imaging

For live cell imaging myoblasts were seeded in 8 well glass slide (Lab-Tek® Chamber Slide™ Coverglass Systems Nunc, Rochester, New York, United States) and were incubated in fusion media for seven days. To identify myotubes cells were stained with anti-NCAM ab (Miltenyi Biotec, Bergisch Gladbach, Germany). For imaging cells were placed in uncoloured DMEM (Gibco, Paisley, UK) and incubated at 37°C and 5% CO_2_ (heating unit, CO_2_ controller and tempcontrol 37-2 from PECON, Erlbach, Germany)_._ Immediately prior to imaging ATTO-495-labeled TAT-labeled specific peptide was added at a final concentration of 10 µM. The dysferlin-peptide distribution within myotubes was observed for 14 hours.

### Laser Wounding Assay

Laser wounding was performed on cultured myotubes as described [35], [36] with slight modifications. Briefly, before laser wounding medium was switched to Tyrode solution (140 mM NaCl, 5 mM KCl, 2 mM MgCl_2_ and 10 mM HEPES, pH 7.2) with 2.5 µM FM 1-43 (Molecular Probes, Invitrogen, Paisley, UK) and 2.5 mM CaCl_2_. Myotubes were wounded by irradiating a 2.5×2.5 µm boundary area of the plasma membrane at 50% maximum power (30-mW argon-laser) for 37 seconds using a Zeiss-LSM 510 META confocal microscope with a 63× oil immersion lens (Carl Zeiss Microimaging GmbH, Jena, Germany). 24 images every 20 seconds were captured after injury. Digital images were processed using the Zeiss LSM Image Browser software. In an area of 10×10 µm directly adjacent to the injury site the changes of fluorescent intensity were calculated with ImageJ.

### Interventional Atomic Force Microscopy

Myotubes were also wounded by interventional atomic force microscopy. These experiments were performed using the Atomic Force Microscope (AFM) NanoWizard II (JPK Instruments, Berlin, Germany) at 37°C with a silicon cantilever (model CSC37 from Mikromasch, Tallinn, Estonia) with a spring constant of 0.30 N/m. Prior to the wounding the cantilever was calibrated using the thermal noise method. The AFM was operated in the manipulation mode. In all experiments 2 µm longitudinal lesions were set with a force set to 40 nN. Videos were obtained with a 40× objective lens using the ProgRes CF microscope camera (Jenoptik, Jena, Germany).

### Protein-protein Interaction

The sequence encoding for the C2E domain of *DYSF* p.L1341P was amplified by PCR using primers to generate XhoI and EcoRI sites at the 5′ and 3′ ends. PCR products were inserted into a His-Tag Vector pRSETA (Invitrogen, Karlsruhe, Germany). Recombinant proteins were over-expressed in BL 21 codon plus (DE3) RIL cells. Proteins were solubilized with Sarcosyl N-LAUROYLSARCOSINE and purified using Ni-NTA columns. Dysferlin specific peptides B1-3 spanning the mutated region of the protein were synthesized by Biosynthan. Protein-binding studies were performed by surface plasmon resonance analysis (Biacore 2000 with CM5 sensor chip; GE Healthcare Europe, Freiburg, Germany).

### RNA Extraction from Cells and cDNA Synthesis from Total RNA

Total RNA was isolated according to the NucleoSpin® RNA II kit from Macherey-Nagel (Düren, Germany). 5–10 g total RNA of each sample was mixed with 3 µl random hexamers (50 ng/µl). DEPC-treated water was added up to the final volume of 12 µl. Each sample was incubated at 70°C for 10 min for primer hybridisation and chilled on ice for at least 1 min. Samples were briefly centrifuged and 8 µl of the reaction mix, containing 4 µl 5× first strand buffer (Gibco), 2 µl DTT (0.1 M), 1 µl dNTP mix (10 mM), 1 µl RNAse inhibitor (RNasin 40 U), were added. Contents of the tubes were mixed and incubated at 25°C for 5 min. 1 µl (200 U) of Superscript II RT was added before incubating the samples at 42°C for 50 min. cDNA synthesis reaction samples containing random hexamers were first incubated at 25°C for 10 min, followed by 50 min at 42°C. The reaction was inactivated by incubating at 70°C for 15 min and the cDNA was stored at −20°C.

### Quantitative Real-time PCR (qRT-PCR)

To detect and quantify (as absolute number of copies or relative amount when normalized to DNA input or additional normalizing genes), we performed a qRT-PCR.

Total cellular RNA was reverse transcribed into DNA. By using specific primer and FAM-TAMRA labeled probes (Table S1) in a TaqMan® PCR we analysed the expression levels of genes compared to the housekeeping gene *abl*. The qPCR was performed in an Eppendorf realplex^2^ Mastercycler epgradient S instrument (Eppendorf, Hamburg, Germany). 5 µl of TaqMan® Gene Expression Master Mix (Applied Biosystems, Darmstadt, Germany), 0.25 µl of forward and reverse primer, 0.3 µl of the probe, and 0.2 µl H_2_O was added to 5 µl of the cDNA or 5 µl of the cDNA standard. All samples were measured in triplicates. Primer and probes for RT-PCR (TaqMan) analysis were ordered from Tib Molbiol as complete gene expression assay or synthesized by BioTez GmbH (Berlin, Germany) (Table S1). Results were normalized to the housekeeping gene *abl* and analyzed by the ΔΔCt method to give fold induction as compared with untreated control samples. Statistics were performed using the Mann-Whitney-U-Test by Dr. Andreas Busjahn.

## Supporting Information

Figure S1 **Position of dysferlin-peptides used for relocalization experiments.** 10- and 15-mer peptides from the dysferlin sequence were synthesized and coupled to the cell penetrating peptide TAT (YGRKKRRQRRR). Peptides A1–4 and B1–4 (Table 1) represent the amino-acid sequence corresponding to *DYSF* p.G299R in the C2B (red) and p.L1431P in the C2E domain (green) [53].
(TIF)

Figure S2 **Nonsense peptides and peptides A4 and B1 do not relocate mutant dysferlin to the sarcolemma.** C2C12 cells were transfected with either missense-mutated dysferlin cDNA *DYSF* p.G299R (upper lane) or p.L1341P (lower lane). Transfected cells were treated with TAT-labeled dysferlin-peptides. Bar: 10 µm.
(TIF)

Figure S3 **Relocated dysferlin abuts to the basal lamina.** Primary human myotubes carrying the dysferlin missense mutation *DYSF* p.G299R in (**A)** and immortalized human myotubes carrying the *DYSF* p.L1341P mutation in (**B**) were treated with the dysferlin-peptides. Dysferlin was detected by anti-dysferlin ab (left column). Co-staining with WGA as a marker of the basal lamina was performed (middle column). Merge is shown at the right column. Arrows indicate the sarcolemmal reallocation of dysferlin by the 10mer mutant dysferlin-peptides. Bar: 10 µm.
(TIF)

Figure S4 ***DYSF*** **p.L1341P does not stably bind corresponding dysferlin peptides.** Protein binding studies were performed by surface plasmon resonance analysis. As analyte the recombinant C2E domain harboring *DYSF* p.L1341P expressed in E.coli was used. The kinetics of interaction, the rates of association and dissociation between the C2E and the corresponding peptides B1, B2 and B3 were tested. There is no evidence for binding between C2E and dysferlin peptides.
(TIF)

Figure S5 **ER stress is reduced by TAT-labeled dysferlin-peptides.** (**A**) In human myotubes harboring the *DYSF* p.G299R mutation specific peptides reduce the relative gene expression of the ER stress mediators *ATF6* (ATF6), *EIF2AK3* (PERK) and *DDIT3* (CHOP). The nonsense peptide serves as control. Data represent median + SEM, n = 9/group. P values are listed in (**C**). (**B**) The 15mer mutant peptide A4 also effectively reduces ER stress, whereas both wildtype peptides A1 and A3 do not have an equivalent effect on the relative gene expression of *ATF6, EIF2AK3 and DDIT3*. Data represent median + SEM, n = 3/group. (**C**) Supplementary statistics to Fig. 6 and Fig. S5A. The fold induction of each sample condition (treatment with peptide A2 or nonsense peptide) is compared to untreated controls *DYSF* p.G299R. Median ± SEM for each condition is listed and p values are indicated in the table; n = 9/group.
(TIF)

Table S1 **Primers and probes for RT-PCR.**
(DOC)

Video S1 **FM1-43 uptake after laser induced damage in** ***DYSF*** **p.G299R primary human myotubes.** No peptides added.
(MOV)

Video S2 **FM1-43 uptake after laser induced damage in** ***DYSF*** **p.G299R primary human myotubes.** Nonsense peptide added (control peptide).
(MOV)

Video S3 **FM1-43 uptake after laser induced damage in** ***DYSF*** **p.G299R primary human myotubes.** Corresponding peptide A2 added.
(MOV)

Video S4 **FM1-43 uptake after laser induced damage in normal primary human myotubes.**
(MOV)

Video S5 **Sarcolemmal injury in normal primary human myotubes induced by atomic force microscopy.**
(MOV)

Video S6 **Sarcolemmal injury in** ***DYSF*** **p.L1341P primary human myotubes induced by atomic force microscopy.** No peptides added.
(MOV)

Video S7 **Sarcolemmal injury in** ***DYSF*** **p.L1341P primary human myotubes induced by atomic force microscopy.** Corresponding peptide B2 added.
(MOV)

Video S8 **Life cell imaging. ATTO-495-ME-labeled and TAT conjugated dysferlin-peptide B2 in** ***DYSF*** **p.L1341P primary human myotubes.** Time lapse is indicated in the video.
(MOV)

## References

[1] Anderson LV, Davison K, Moss JA, Young C, Cullen MJ, et al (1999) Dysferlin is a plasma membrane protein and is expressed early in human development. Hum Mol Genet 8: 855–861. 10196375 10.1093/hmg/8.5.855

[2] Bansal D, Miyake K, Vogel SS, Groh S, Chen CC, et al (2003) Defective membrane repair in dysferlin-deficient muscular dystrophy. Nature 423: 168–172. 12736685 10.1038/nature01573

[3] Bashir R, Britton S, Strachan T, Keers S, Vafiadaki E, et al (1998) A gene related to Caenorhabditis elegans spermatogenesis factor fer-1 is mutated in limb-girdle muscular dystrophy type 2B. Nat Genet 20: 37–42. 9731527 10.1038/1689

[4] Liu J, Aoki M, Illa I, Wu C, Fardeau M, et al (1998) Dysferlin, a novel skeletal muscle gene, is mutated in Miyoshi myopathy and limb girdle muscular dystrophy. Nat Genet 20: 31–36. 9731526 10.1038/1682

[5] Krahn M, Beroud C, Labelle V, Nguyen K, Bernard R, et al (2009) Analysis of the DYSF mutational spectrum in a large cohort of patients. Hum Mutat 30: E345–375. 18853459 10.1002/humu.20910

[6] Wenzel K, Carl M, Perrot A, Zabojszcza J, Assadi M, et al (2006) Novel sequence variants in dysferlin-deficient muscular dystrophy leading to mRNA decay and possible C2-domain misfolding. Hum Mutat 27: 599–600. 10.1002/humu.9424 16705711

[7] Fujita E, Kouroku Y, Isoai A, Kumagai H, Misutani A, et al (2007) Two endoplasmic reticulum-associated degradation (ERAD) systems for the novel variant of the mutant dysferlin: ubiquitin/proteasome ERAD(I) and autophagy/lysosome ERAD(II). Hum Mol Genet 16: 618–629. 17331981 10.1093/hmg/ddm002

[8] Spuler S, Carl M, Zabojszcza J, Straub V, Bushby K, et al (2008) Dysferlin-deficient muscular dystrophy features amyloidosis. Ann Neurol 63: 323–328. 18306167 10.1002/ana.21309

[9] Evesson FJ, Peat RA, Lek A, Brilot F, Lo HP, et al (2010) Reduced plasma membrane expression of dysferlin mutants is attributed to accelerated endocytosis via a syntaxin-4-associated pathway. J Biol Chem 285: 28529–28539. 20595382 10.1074/jbc.M110.111120 PMC2937879

[10] Bartolini M, Andrisano V (2010) Strategies for the inhibition of protein aggregation in human diseases. Chembiochem 11: 1018–1035. 20401887 10.1002/cbic.200900666

[11] Jones D (2010) Modifying protein misfolding. Nat Rev Drug Discov 9: 825–827. 21030987 10.1038/nrd3316

[12] Selcen D, Stilling G, Engel AG (2001) The earliest pathologic alterations in dysferlinopathy. Neurology 56: 1472–1481. 11402103 10.1212/wnl.56.11.1472

[13] Ellis J (1987) Proteins as molecular chaperones. Nature 328: 378–379. 3112578 10.1038/328378a0

[14] Hammond C, Helenius A (1995) Quality control in the secretory pathway. Curr Opin Cell Biol 7: 523–529. 7495572 10.1016/0955-0674(95)80009-3

[15] Ushioda R, Hoseki J, Araki K, Jansen G, Thomas DY, et al (2008) ERdj5 is required as a disulfide reductase for degradation of misfolded proteins in the ER. Science 321: 569–572. 18653895 10.1126/science.1159293

[16] Kozutsumi Y, Segal M, Normington K, Gething MJ, Sambrook J (1988) The presence of malfolded proteins in the endoplasmic reticulum signals the induction of glucose-regulated proteins. Nature 332: 462–464. 3352747 10.1038/332462a0

[17] Dorner AJ, Wasley LC, Kaufman RJ (1992) Overexpression of GRP78 mitigates stress induction of glucose regulated proteins and blocks secretion of selective proteins in Chinese hamster ovary cells. EMBO J 11: 1563–1571. 1373378 10.1002/j.1460-2075.1992.tb05201.x PMC556605

[18] Gulow K, Bienert D, Haas IG (2002) BiP is feed-back regulated by control of protein translation efficiency. J Cell Sci 115: 2443–2452. 12006628 10.1242/jcs.115.11.2443

[19] Leborgne-Castel N, Jelitto-Van Dooren EP, Crofts AJ, Denecke J (1999) Overexpression of BiP in tobacco alleviates endoplasmic reticulum stress. Plant Cell 11: 459–470. 10072404 10.1105/tpc.11.3.459 PMC144191

[20] Little E, Lee AS (1995) Generation of a mammalian cell line deficient in glucose-regulated protein stress induction through targeted ribozyme driven by a stress-inducible promoter. J Biol Chem 270: 9526–9534. 7721881

[21] Haze K, Yoshida H, Yanagi H, Yura T, Mori K (1999) Mammalian transcription factor ATF6 is synthesized as a transmembrane protein and activated by proteolysis in response to endoplasmic reticulum stress. Mol Biol Cell 10: 3787–3799. 10564271 10.1091/mbc.10.11.3787 PMC25679

[22] Wang Y, Shen J, Arenzana N, Tirasophon W, Kaufman RJ, et al (2000) Activation of ATF6 and an ATF6 DNA binding site by the endoplasmic reticulum stress response. J Biol Chem 275: 27013–27020. 10856300 10.1074/jbc.M003322200

[23] Brostrom CO, Brostrom MA (1998) Regulation of translational initiation during cellular responses to stress. Prog Nucleic Acid Res Mol Biol 58: 79–125. 9308364 10.1016/s0079-6603(08)60034-3

[24] Prostko CR, Brostrom MA, Malara EM, Brostrom CO (1992) Phosphorylation of eukaryotic initiation factor (eIF) 2 alpha and inhibition of eIF-2B in GH3 pituitary cells by perturbants of early protein processing that induce GRP78. J Biol Chem 267: 16751–16754. 1512215

[25] Matsumoto M, Minami M, Takeda K, Sakao Y, Akira S (1996) Ectopic expression of CHOP (GADD153) induces apoptosis in M1 myeloblastic leukemia cells. FEBS Lett 395: 143–147. 8898082 10.1016/0014-5793(96)01016-2

[26] Ron D, Habener JF (1992) CHOP, a novel developmentally regulated nuclear protein that dimerizes with transcription factors C/EBP and LAP and functions as a dominant-negative inhibitor of gene transcription. Genes Dev 6: 439–453. 1547942 10.1101/gad.6.3.439

[27] Zinszner H, Kuroda M, Wang X, Batchvarova N, Lightfoot RT, et al (1998) CHOP is implicated in programmed cell death in response to impaired function of the endoplasmic reticulum. Genes Dev 12: 982–995. 9531536 10.1101/gad.12.7.982 PMC316680

[28] Askanas V, Engel WK, Nogalska A (2009) Inclusion body myositis: a degenerative muscle disease associated with intra-muscle fiber multi-protein aggregates, proteasome inhibition, endoplasmic reticulum stress and decreased lysosomal degradation. Brain Pathol 19: 493–506. 19563541 10.1111/j.1750-3639.2009.00290.x PMC8094750

[29] Clemen CS, Tangavelou K, Strucksberg KH, Just S, Gaertner L, et al (2010) Strumpellin is a novel valosin-containing protein binding partner linking hereditary spastic paraplegia to protein aggregation diseases. Brain 133: 2920–2941. 20833645 10.1093/brain/awq222

[30] Gueneau L, Bertrand AT, Jais JP, Salih MA, Stojkovic T, et al (2009) Mutations of the FHL1 gene cause Emery-Dreifuss muscular dystrophy. Am J Hum Genet 85: 338–353. 19716112 10.1016/j.ajhg.2009.07.015 PMC2771595

[31] Selcen D (2011) Myofibrillar myopathies. Neuromuscul Disord 21: 161–171. 21256014 10.1016/j.nmd.2010.12.007 PMC3052736

[32] Sawant R, Torchilin V (2010) Intracellular transduction using cell-penetrating peptides. Mol Biosyst 6: 628–640. 20237640 10.1039/b916297f

[33] Fittipaldi A, Ferrari A, Zoppe M, Arcangeli C, Pellegrini V, et al (2003) Cell membrane lipid rafts mediate caveolar endocytosis of HIV-1 Tat fusion proteins. J Biol Chem 278: 34141–34149. 12773529 10.1074/jbc.M303045200

[34] Lionetti V, Fittipaldi A, Agostini S, Giacca M, Recchia FA, et al (2009) Enhanced caveolae-mediated endocytosis by diagnostic ultrasound in vitro. Ultrasound Med Biol 35: 136–143. 18950933 10.1016/j.ultrasmedbio.2008.07.011

[35] Cai C, Weisleder N, Ko JK, Komazaki S, Sunada Y, et al (2009) Membrane repair defects in muscular dystrophy are linked to altered interaction between MG53, caveolin-3, and dysferlin. J Biol Chem 284: 15894–15902. 19380584 10.1074/jbc.M109.009589 PMC2708885

[36] Marg A, Schoewel V, Timmel T, Schulze A, Shah C, et al (2012) Sarcolemmal Repair Is a Slow Process and Includes EHD2. Traffic 2012 13(9): 1286–1294.37. 10.1111/j.1600-0854.2012.01386.x 22679923

[37] Jung SH, Park D, Park JH, Kim YM, Ha KS (2010) Molecular imaging of membrane proteins and microfilaments using atomic force microscopy. Exp Mol Med 42: 597–605. 20689364 10.3858/emm.2010.42.9.064 PMC2947017

[38] Yum K, Wang N, Yu MF (2010) Nanoneedle: a multifunctional tool for biological studies in living cells. Nanoscale 2: 363–372. 20644817 10.1039/b9nr00231f

[39] Calamini B, Silva MC, Madoux F, Hutt DM, Khanna S, et al (2012) Small-molecule proteostasis regulators for protein conformational diseases. Nat Chem Biol 8: 185–196. 10.1038/nchembio.763 PMC3262058 22198733

[40] Cormet-Boyaka E, Hong JS, Berdiev BK, Fortenberry JA, Rennolds J, et al (2009) A truncated CFTR protein rescues endogenous DeltaF508-CFTR and corrects chloride transport in mice. FASEB J 23: 3743–3751. 19620404 10.1096/fj.08-127878 PMC2775001

[41] Cormet-Boyaka E, Jablonsky M, Naren AP, Jackson PL, Muccio DD, et al (2004) Rescuing cystic fibrosis transmembrane conductance regulator (CFTR)-processing mutants by transcomplementation. Proc Natl Acad Sci U S A 101: 8221–8226. 15141088 10.1073/pnas.0400459101 PMC419584

[42] Xu L, Pallikkuth S, Hou Z, Mignery GA, Robia SL, et al (2011) Dysferlin forms a dimer mediated by the C2 domains and the transmembrane domain in vitro and in living cells. PLoS One 6: e27884. 22110769 10.1371/journal.pone.0027884 PMC3215728

[43] Lostal W, Bartoli M, Bourg N, Roudaut C, Bentaib A, et al (2010) Efficient recovery of dysferlin deficiency by dual adeno-associated vector-mediated gene transfer. Hum Mol Genet 19: 1897–1907. 20154340 10.1093/hmg/ddq065

[44] Azakir BA, Di Fulvio S, Kinter J, Sinnreich M (2012) Proteasomal inhibition restores biological function of Missense mutated dysferlin in patient derived muscle cells. J Biol Chem 287: 10344–10354. 22318734 10.1074/jbc.M111.329078 PMC3323038

[45] Ling YH, Liebes L, Ng B, Buckley M, Elliott PJ, et al (2002) PS-341, a novel proteasome inhibitor, induces Bcl-2 phosphorylation and cleavage in association with G2-M phase arrest and apoptosis. Mol Cancer Ther 1: 841–849. 12492117

[46] Cavaletti G, Gilardini A, Canta A, Rigamonti L, Rodriguez-Menendez V, et al (2007) Bortezomib-induced peripheral neurotoxicity: a neurophysiological and pathological study in the rat. Exp Neurol 204: 317–325. 17214983 10.1016/j.expneurol.2006.11.010

[47] Fukai T (2007) Targeting proteasome worsens atherosclerosis. Circ Res 101: 859–861. 17967795 10.1161/CIRCRESAHA.107.164020

[48] Herrmann J, Saguner AM, Versari D, Peterson TE, Chade A, et al (2007) Chronic proteasome inhibition contributes to coronary atherosclerosis. Circ Res 101: 865–874. 17823377 10.1161/CIRCRESAHA.107.152959

[49] Richardson PG, Barlogie B, Berenson J, Singhal S, Jagannath S, et al (2003) A phase 2 study of bortezomib in relapsed, refractory myeloma. N Engl J Med 348: 2609–2617. 12826635 10.1056/NEJMoa030288

[50] Richardson PG, Briemberg H, Jagannath S, Wen PY, Barlogie B, et al (2006) Frequency, characteristics, and reversibility of peripheral neuropathy during treatment of advanced multiple myeloma with bortezomib. J Clin Oncol 24: 3113–3120. 16754936 10.1200/JCO.2005.04.7779

[51] Belanto JJ, Diaz-Perez SV, Magyar CE, Maxwell MM, Yilmaz Y, et al (2010) Dexamethasone induces dysferlin in myoblasts and enhances their myogenic differentiation. Neuromuscul Disord 20: 111–121. 20080405 10.1016/j.nmd.2009.12.003 PMC2856642

[52] Philippi S, Bigot A, Marg A, Mouly V, Spuler S, et al. (2012) Dysferlin-deficient immortalized human myoblasts and myotubes as a useful tool to study dysferlinopathy. PLoS Currents Muscular Dystrophy. 2012 Feb 28 [last modified: 2012 Mar 26] doi: 10.1371/currents.RRN1298. 10.1371/currents.RRN1298 PMC3274833 22367358

[53] Anderson LV, Harrison RM, Pogue R, Vafiadaki E, Pollitt C, et al (2000) Secondary reduction in calpain 3 expression in patients with limb girdle muscular dystrophy type 2B and Miyoshi myopathy (primary dysferlinopathies). Neuromuscul Disord 10: 553–559. 11053681 10.1016/s0960-8966(00)00143-7

