# Supplementary figures and images for: Dysferlin-Peptides Reallocate Mutated Dysferlin Thereby Restoring Function
Source: PLoS One. 2012 Nov 20;7(11):e49603. doi: 10.1371/journal.pone.0049603 (PMC3502493; doi:10.1371/journal.pone.0049603)

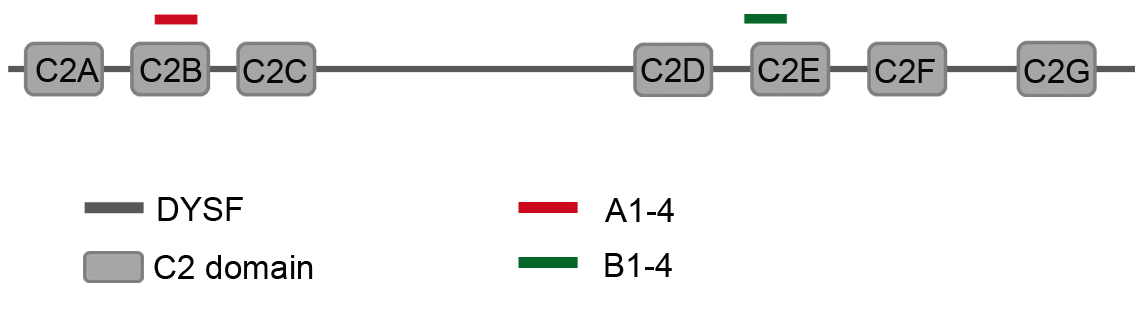

Supplement: Figure S1 — Position of dysferlin-peptides used for relocalization experiments. 10- and 15-mer peptides from the dysferlin sequence were synthesized and coupled to the cell penetrating peptide TAT (YGRKKRRQRRR). Peptides A1–4 and B1–4 (Table 1) represent the amino-acid sequence corresponding to DYSF p.G299R in the C2B (red) and p.L1431P in the C2E domain (green) [53]. (TIF) [file pone.0049603.s001.tif]

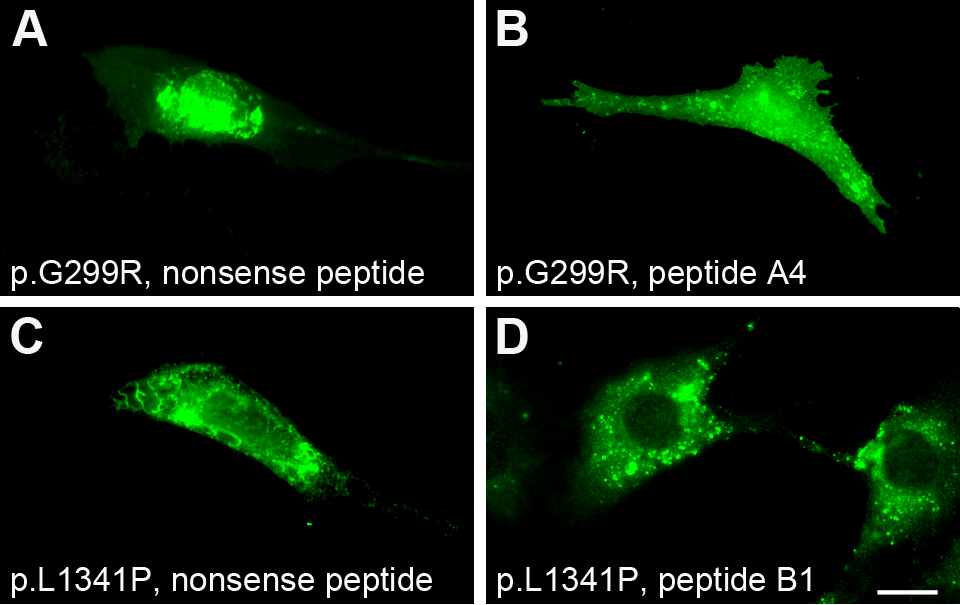

Supplement: Figure S2 — Nonsense peptides and peptides A4 and B1 do not relocate mutant dysferlin to the sarcolemma. C2C12 cells were transfected with either missense-mutated dysferlin cDNA DYSF p.G299R (upper lane) or p.L1341P (lower lane). Transfected cells were treated with TAT-labeled dysferlin-peptides. Bar: 10 µm. (TIF) [file pone.0049603.s002.tif]

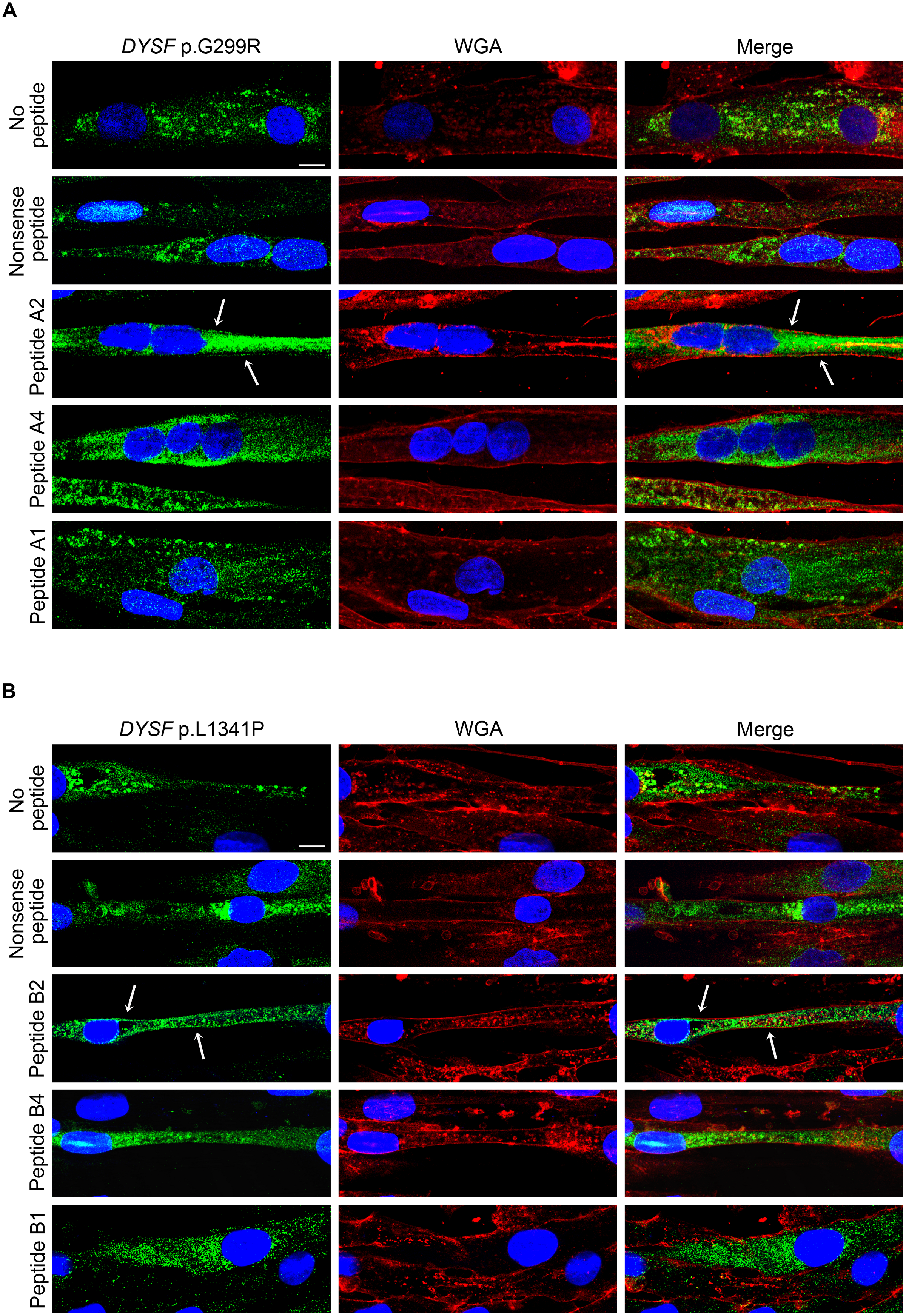

Supplement: Figure S3 — Relocated dysferlin abuts to the basal lamina. Primary human myotubes carrying the dysferlin missense mutation DYSF p.G299R in (A) and immortalized human myotubes carrying the DYSF p.L1341P mutation in (B) were treated with the dysferlin-peptides. Dysferlin was detected by anti-dysferlin ab (left column). Co-staining with WGA as a marker of the basal lamina was performed (middle column). Merge is shown at the right column. Arrows indicate the sarcolemmal reallocation of dysferlin by the 10mer mutant dysferlin-peptides. Bar: 10 µm. (TIF) [file pone.0049603.s003.tif]

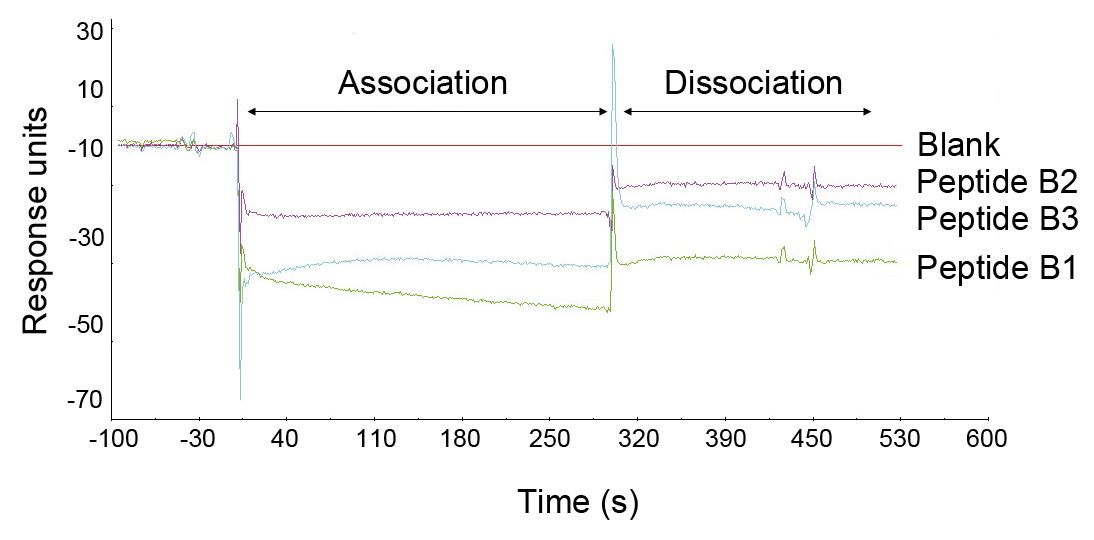

Supplement: Figure S4 — DYSF p.L1341P does not stably bind corresponding dysferlin peptides. Protein binding studies were performed by surface plasmon resonance analysis. As analyte the recombinant C2E domain harboring DYSF p.L1341P expressed in E.coli was used. The kinetics of interaction, the rates of association and dissociation between the C2E and the corresponding peptides B1, B2 and B3 were tested. There is no evidence for binding between C2E and dysferlin peptides. (TIF) [file pone.0049603.s004.tif]

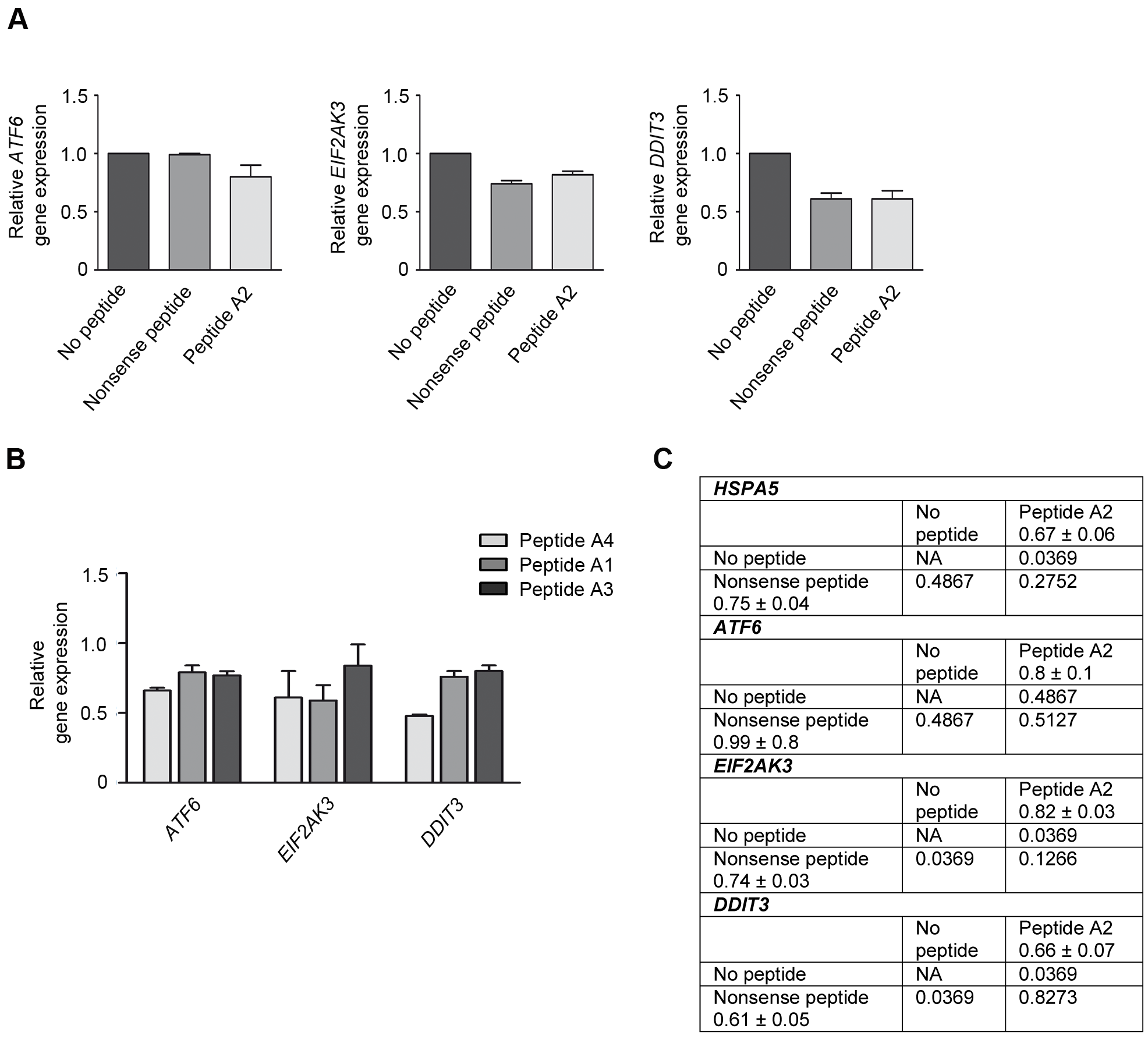

Supplement: Figure S5 — ER stress is reduced by TAT-labeled dysferlin-peptides. (A) In human myotubes harboring the DYSF p.G299R mutation specific peptides reduce the relative gene expression of the ER stress mediators ATF6 (ATF6), EIF2AK3 (PERK) and DDIT3 (CHOP). The nonsense peptide serves as control. Data represent median + SEM, n = 9/group. P values are listed in (C). (B) The 15mer mutant peptide A4 also effectively reduces ER stress, whereas both wildtype peptides A1 and A3 do not have an equivalent effect on the relative gene expression of ATF6, EIF2AK3 and DDIT3. Data represent median + SEM, n = 3/group. (C) Supplementary statistics to Fig. 6 and Fig. S5A. The fold induction of each sample condition (treatment with peptide A2 or nonsense peptide) is compared to untreated controls DYSF p.G299R. Median ± SEM for each condition is listed and p values are indicated in the table; n = 9/group. (TIF) [file pone.0049603.s005.tif]
