# Supplementary material for: Dysferlin-Peptides Reallocate Mutated Dysferlin Thereby Restoring Function
Source: PLoS One. 2012 Nov 20;7(11):e49603. doi: 10.1371/journal.pone.0049603 (PMC3502493; doi:10.1371/journal.pone.0049603)
Supplement: Table S1 — Primers and probes for RT-PCR. (DOC) [file pone.0049603.s006.doc]

**Table S1**: Primers and probes for RT-PCR

| **Name** | **Forward primer** | **Reverse primer** | **Probe** |
| --- | --- | --- | --- |
| ***ATF6*** | 5‘-TCT CTT TgC TgA ACT Cgg TTA TTT C-3‘ | 5‘-AAT TgT TTT CAT ACg TCT CAT TTg CT-3‘ | 6FAM-CAg ACA CTg ATg AgC TgC AAT Tgg AA--TMR |
| ***DDIT3/ CHOP*** | 5‘-ggA AAT gAA gAg gAA gAA TCA AAA AT-3‘ | 5‘-gTT CTg gCT CCT CCT CAg TCA-3‘ | 6FAM-TTC ACC ACT CTT gAC CCT gCT TCT CTg g--TMR |
| ***HSPA5/ GRP78/BiP*** | 5‘-gCA ACC AAA gAC gCT ggA A-3‘ | 5‘-TgC CgT Agg CTC gTT gAT g-3‘ | 6FAM-ATT gCT ggC CTA AAT gTT ATg Agg A--TMR |
| ***EIF2AK3/ PERK*** | 5‘-gCA AAC CAg Agg TAT TTg ggA A -3‘ | 5‘-ggT CTT ggTCCC ACT ggA AgA-3‘ | 6FAM-ATg ATC ATT CCT TCC CTg gAT ggA gCC--TMR |
| ***ABL*** | 5‘-Tgg AgA TAA CAC TCT AAg CAT AAC TAA Agg T-3‘ | 5‘-gAT gTA gTT gCT Tgg gAC CCA-3‘ | 6FAM-CCA TTT TTg gTT Tgg gCT TCA CAC CAT T--TMR |
